# The impact of COVID-19 on general vaccine acceptance in low- and middle-income countries: a systematic review

**DOI:** 10.3389/fpubh.2026.1764389

**Published:** 2026-02-23

**Authors:** Gloria Lihemo, Madeleine Blunt, Ibrahim Dadari, Talya Underwood, Andres Esteban Ochoa Toasa, Alina Velias, Kathryn L. Hopkins, Angus Thomson, Robert Kanwagi, Amaya Gillespie, Deepa Risal Pokharel, Smita Singh

**Affiliations:** 1 United Nations Children's Fund (UNICEF) HQ, New York, NY, United States; 2 Department of Psychology and Behavioural Science, London School of Economics and Political Science, London, United Kingdom; 3 Department of International Health, Johns Hopkins Bloomberg School of Public Health, Baltimore, MD, United States; 4 Consultant to Sabin Vaccine Institute, Northwich, United Kingdom; 5 Sabin Vaccine Institute, Washington, DC, United States; 6 Irimi, Lyon, France; 7 London School of Hygiene & Tropical Medicine, London, United Kingdom; 8 GAVI, the Vaccine Alliance, Geneva, Switzerland

**Keywords:** COVID-19, systematic review, vaccination, vaccine, vaccine acceptance, vaccine confidence

## Abstract

**Background:**

The COVID-19 pandemic caused a major decline in childhood vaccination, especially in low- and middle-income countries (LMICs). However, its specific impact on vaccine hesitancy in the immediate post-pandemic years, particularly toward non-COVID-19 vaccines, remains unclear. Understanding the social and behavioral factors influencing vaccine acceptance following a public health emergency such as the COVID-19 pandemic is critical to improving immunization coverage. This systematic review examined the impact of the COVID-19 pandemic on general vaccine acceptance in LMICs to inform strategies to improve vaccine uptake.

**Methods:**

This systematic review assessed people's thinking and feeling, motivations, practical issues, and social processes around vaccination, conceptualized by the World Health Organization's Behavioural and Social Drivers framework. Studies were included if they were interventional or observational in design, examined the impact of the COVID-19 pandemic on vaccine hesitancy or acceptance for non-COVID-19 vaccines, and were published in English between 2020 and 2023.

**Results:**

A total of 23 studies were included in the review, with most studies conducted in middle-income settings and focused on healthcare workers or parents/caregivers of children. Findings belonging to the “Thinking and Feeling” category were the most commonly reported in 91% (*n* = 21/23) of studies. Over half (61%) of studies reported findings relating to the ‘Motivation' construct, while 43% of studies reported outcomes related to ‘Practical Issues' and ‘Social Processes'. Studies reported both increases and decreases in vaccine hesitancy and intention to vaccinate due to the pandemic. Overall, studies most commonly reported that the COVID-19 pandemic had a negative or neutral effect on attitudes, intentions, and actions regarding vaccine acceptance.

**Conclusion:**

This systematic review illustrates how the COVID-19 pandemic influenced vaccine acceptance and decision-making in complex, context-dependent ways, impacting people's thinking and feeling, motivations, practical issues, and social processes around vaccination. The findings highlight the need to understand the specific drivers of vaccine acceptance to design more effective, targeted strategies to improve immunization uptake. The insights from this study can be used to inform evidence-based vaccination catch-up strategies to regain pandemic losses and mitigate factors that deter individuals from seeking vaccination.

## Introduction

The COVID-19 pandemic has resulted in the largest decline in childhood vaccinations in nearly three decades (1). Over 25 million children missed out on lifesaving vaccinations in 2021, compared with 19 million in 2019 (2). The majority of these children are from low- and middle-income countries (LMICs), where the pandemic resulted in widespread disruption of health services including vaccination programs (2, 3). While the pandemic had a negative and lasting impact on vaccine uptake across immunization programs, its impact on hesitancy toward non-COVID-19 vaccines has been less clear.

Vaccine hesitancy was recently redefined by the World Health Organization's Strategic Advisory Group of Experts on Immunization (SAGE) as “the motivational state of being conflicted about, or opposed to, getting vaccinated” (4). There has been limited research on how the COVID-19 pandemic affected hesitancy toward other vaccines, such as measles or polio vaccines. One study conducted in the UK assessed general vaccine confidence using an adapted version of the WHO SAGE Vaccine Hesitancy Scale (5). In this context, confidence is defined as trust in vaccines, the healthcare system administering the vaccine, and the policy-makers promoting or mandating the vaccine (5). The study found a significant decrease in vaccine confidence across all demographic groups from pre- to post-pandemic, although 76% of respondents self-reported no change or an increase in confidence over this period (5). A previous systematic review examining the impact of the COVID-19 pandemic on vaccine coverage, services, and confidence found consistent declines in vaccination rates, but did not identify any trends for vaccine confidence, perhaps reflecting reduced access to services rather than a substantial shift in attitudes alone (6). The impact of the COVID-19 pandemic on vaccine confidence and acceptance therefore remains unclear, particularly in terms of the social and behavioral factors driving vaccine acceptance.

Understanding the social and behavioral determinants of immunization is critical to promoting and sustaining high vaccination coverage (7). Reluctance to adhere to preventative measures, such as vaccination, has been a recurring phenomenon during disease outbreaks, including the COVID-19 pandemic (8–10). As such, it is important to understand how the COVID-19 pandemic affected people's behaviors and decision-making for both current and future vaccinations.

Consequently, this systematic review set out to provide the first comprehensive assessment of the COVID-19 pandemic's impact on the acceptance of both childhood routine and adult vaccination in LMICs during the pandemic and in the immediate post-pandemic period. Specifically, the review examined how perceptions about COVID-19 vaccination and the broader health system affect people's current and future perceptions about vaccination in general. Acceptance of vaccination was defined in this review as the interplay of different constructs shaped by belief in the proper development and trial of vaccines, trust in the authorities distributing them, and the trade-offs of the disease risk and benefits (11).

**Figure 1:**
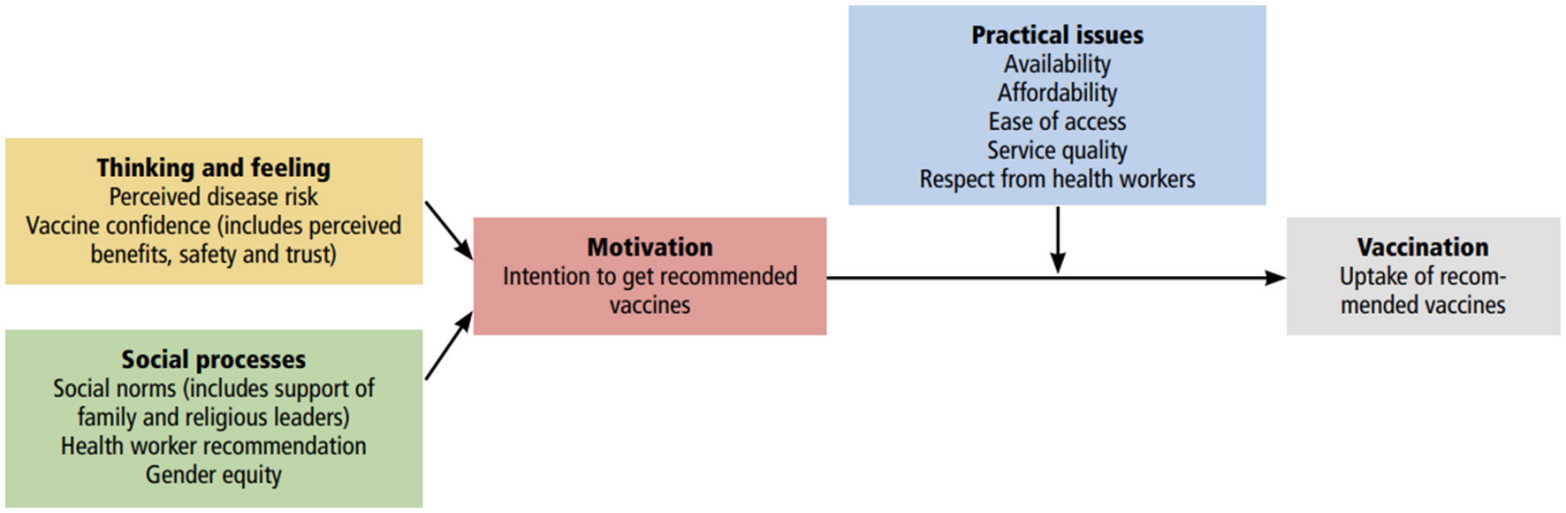
Behavioural and social drivers framework. Reproduced from Understanding the behavioural and social drivers of vaccine uptake WHO position paper – May 2022 (4) under the terms of the Creative Commons Attribution-NonCommercial-ShareAlike 3.0 IGO license: https://creativecommons.org/licenses/by-nc-sa/3.0/igo/deed.en.

The systematic review was conceptualized by the WHO Behavioural and Social Drivers (BeSD) framework for vaccination uptake (4). The BeSD framework was selected as it offers a structured approach to understanding the interplay of social and behavioral factors that underpin vaccine acceptance. The framework is also recommended by WHO's SAGE and validated for use in LMICs (4). It groups vaccination beliefs and experiences into four modifiable benchmarks (Figure 1) [4]: a) *Thinking and feeling*: the cognitive and emotional reaction to vaccination including confidence in vaccination; b) *Social processes*: vaccination norms including acceptance of vaccination recommendation; c) *Motivation*: willingness and intent to vaccinate including vaccine hesitancy; and d) *Practical issues*: physical barriers like access and opportunity costs of vaccination including people's experiences while seeking vaccination.

Through application of the BeSD framework, the systematic review provides an in-depth insight into the multifaceted effects of the COVID-19 pandemic on determinants of vaccine acceptance and uptake. These insights can support more effective vaccination catch-up strategies to regain pandemic losses and help mitigate factors that deter individuals from seeking vaccination.

## Methods

This systematic review was conducted in accordance with the Preferred Reporting Items for Systematic Reviews and Meta-Analyses (PRISMA) 2020 guidelines. PRISMA guidelines were followed at each stage of the review, including the formulation of research objectives, development of eligibility criteria, comprehensive literature search, transparent reporting of study selection, data extraction, critical appraisal, and synthesis of findings. The study selection process was documented in a PRISMA flow diagram. The review protocol was registered on the International Prospective Register of Systematic Reviews (PROSPERO) under CRD42023492956 (12).

### Study objectives

The study set out to evaluate the impact of the COVID-19 pandemic on acceptance of other vaccines in LMICs, in terms of people's thinking and feeling, motivations, practical issues, and social processes around vaccination, during the pandemic and immediate post-pandemic period up to November 2023.

### Inclusion/exclusion criteria

The inclusion and exclusion criteria followed the Population, Intervention, Comparison, and Outcome (PICO) model as described below. This information was also used to define the search strategy:

- Participants-populations in LMICs;
- Interventions-that evaluate different categories of vaccine acceptance (thoughts and feelings, motivations, practical factors, social processes, trust);
- Comparison-of increase or decrease in people's perceptions of and acceptance of vaccination before, during, and after the COVID-19 pandemic; and
- Outcome-synthesis of study outcomes on perceptions and acceptance of non COVID-19 vaccination before, during, and after the COVID-19 pandemic.

We included only English-language materials published between 2020 and 2023. Eligible studies included both interventional and observational designs that described or attempted to measure the effect of COVID-19 on vaccine hesitancy and/or acceptance. We excluded systematic reviews, protocols, editorials, letters, case reports, case studies, commentaries, opinion pieces, narrative reviews, and clinical guidelines. Studies that did not assess routine immunization hesitancy/acceptance were also excluded. This included, for example, studies that focused solely on vaccination against COVID-19, or studies that focused on immunization supply, coverage, or uptake rather than addressing vaccine hesitancy/acceptance behaviorally. As this study focused on LMICs, we also excluded studies that were conducted only in high-income countries. Studies were permitted if they included data on LMICs and high-income countries, if the data on LMICs could be extracted separately. The inclusion/exclusion criteria are further described in Table 1.

**Table 1:** Inclusion/exclusion criteria.

**Criteria**	**Inclusion**	**Exclusion**
Population	Low- and middle-income countries	High-income countries only
Intervention	COVID-19 pandemic response COVID-19 vaccination delivery COVID-19 prevention strategies	COVID-19 vaccine clinical trials
Outcome	Immunization delivery Immunization programs Immunization hesitancy Immunization acceptance	COVID-19 immunization hesitancy COVID-19 immunization acceptance Immunization coverage only Immunization uptake only Immunization infrastructure/supply
Study characteristics	Interventional study or observational study focused on the effect of COVID-19 on vaccine hesitancy/acceptance	Systematic review Scoping reviews Meta analysis Protocol Editorial Letters Case report Case study Commentary Opinion piece Narrative review Clinical guidelines Animal studies Withdrawn Erratum/correction
Language	English	

### Search strategy

Search terms were developed and revised with input from subject matter experts and an information specialist from the London School of Economics and Political Science (LSE). We conducted our search using MEDLINE (Ovid), Embase, and Global Health databases, chosen for their broad coverage of global health sciences and behavioral research and literature relevant to LMICs. The following keywords (and their synonyms) were used: vaccination hesitancy, low and middle income countries, and COVID-19. The full search strategy is included in Supplementary material S1.

### Study selection and screening

We conducted the database searches on 7 November 2023. Studies were screened by three study team members, in line with Cochrane guidance and to allow for transparency and minimize bias. A total of 10,616 papers were identified from the three databases (Embase = 4,613; MEDLINE = 3,562; and Global Health = 2,441). Search terms were contextualized for each database and uploaded to the Covidence systematic review tool (Melbourne, Australia). In total, 3,950 duplicate publications were identified and removed.

**Figure 2:**
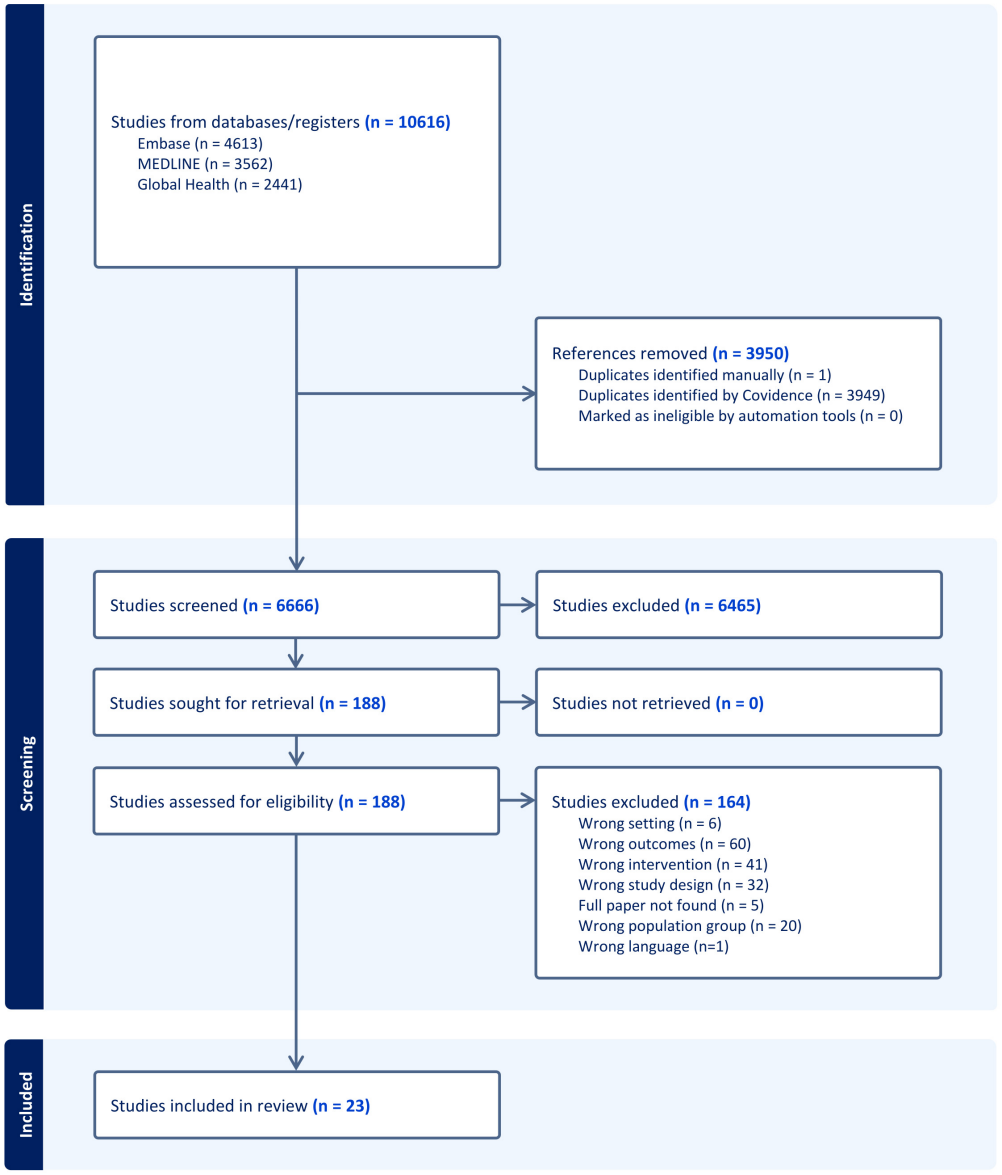
PRISMA diagram.

The titles and abstracts of the remaining 6,666 articles were assessed independently for inclusion criteria by two study team members and 6,465 studies were initially excluded as they did not meet the inclusion criteria. Inclusion/exclusion decisions of both team members were in agreement for 92% of articles. A third study team member reviewed the misaligned articles (*n* = 58/188) and resolved the conflicts. The screening process identified 201 articles for full-text review. The same process for screening and conflict resolution was used to identify 23 articles, which met the criteria for inclusion in this study. The review process is depicted in Figure 2.

### Data extraction

Covidence (Melbourne, Australia) was used to manage the data extraction and quality assessment processes. In addition to identifying study outcomes, extraction focused on data that could be categorized using the BeSD framework (4), as well as sociodemographic information, including study populations, geographical settings, and type of vaccines. Two study members reviewed and extracted relevant data for each article.

Due to the variability of studies in terms of methodology, the Mixed Methods Appraisal Tool (MMAT) was used to appraise study quality (13). The MMAT is designed to assess the methodological quality of qualitative, quantitative, and mixed-methods studies. The tool contains five core criteria tailored to each study design, enabling evaluation of methodological quality across diverse study types.

Two reviewers assessed the studies for quality. The included studies were evaluated based on the 27 questions of the MMAT tool, according to questions relevant for the different study types. Details of the quality assessment questionnaire, along with questions and answers, are provided in Supplementary material S2. All eligible studies were included in the analysis regardless of their MMAT score, as recommended by the tool. Findings from the MMAT appraisal were summarized using a descriptive star-based system as outlined in guidelines on reporting findings from the MMAT tool (14).

### Assessment framework

The impact of the COVID-19 pandemic on vaccine acceptance was assessed according to the aforementioned WHO BeSD framework. Under the “Thinking and Feeling” category, findings/outcomes were categorized into two constructs: “perceived disease risk” and “vaccine confidence”. The former included factors related to perceptions of likelihood for infection and/or the severity of the perceived infection. Outcomes relating to the “Motivation” category were categorized around the intersecting constructs of intent, willingness, acceptance, and hesitancy. Practical factors were considered in terms of the physical and operational determinants that impacted acceptance of vaccination. This category included constructs such as vaccine availability, affordability, ease of access, service quality, and respect from healthcare providers. While structural-based constructs such as lockdowns were excluded from the analysis, practical barriers such as stock-outs and vaccine availability were included as they directly influence acceptance and are a component of the BeSD framework. Under social processes, the constructs examined included “social norms” defined as perceptions of others in one's network with regard to vaccination, “health worker recommendation” for vaccination and “gender equity”.

### Data analysis

Outcomes relating to the impact of the COVID-19 pandemic on the acceptance of other vaccines in LMICs were reported according to the aforementioned BeSD categories and tallied quantitively by category and construct. The numbers of quantitative and qualitative outcomes were also recorded. Studies were also categorized in terms of whether they reported a positive, negative, neutral or mixed effect of the COVID-19 pandemic on vaccine acceptance, in terms of reported attitudes, intentions, and actions. Each study was classified according to the aforementioned categories, with findings tallied quantitatively and presented in a harvest plot. Specifically, studies were classified based on the authors' reported results and narrative conclusions regarding the impact of the pandemic on vaccine acceptance. A study was coded as positive if the findings indicated an increase in measures of vaccine acceptance, negative if an overall decrease was reported, and mixed if both positive and negative effects were observed across outcomes, subpopulations, or time points within the same study. Findings were recorded as neutral when there was no overall change reported in vaccine acceptance or where the study did not measure change in vaccine acceptance (e.g., if it was a qualitative study). Studies were coded as unclear when results were ambiguous or insufficient to support a clear directional classification. Two team members classified the studies based on the criteria. Conflicting results were to be resolved through discussion; however, team members were in agreement for all 23 studies. A formal meta-analysis was not feasible due to the heterogeneity of study designs, measures, and outcomes.

## Results

### Study characteristics

#### Geographical scope and income-level

The review included studies from all regions of the world (Supplementary Table S1). The majority focused on individual countries, while a few encompassed multiple countries based on a regional or income group classification. Among the countries represented, Türkiye had the highest number of studies (*n* = 5), followed by Nigeria (*n* = 3). Other individual study countries included Rwanda, India, Ghana, Jordan, Kenya, Lebanon, Malawi, Colombia, and Indonesia. Additional studies looked at multiple countries in sub-Saharan African (15), the Middle Eastern, Northern Africa, and South Asia regions (16), and the UK, France, Germany, Italy, Brazil, Argentina, and Australia (with data only included for LMICs within these studies) (17).

Most of the studies were conducted in middle-income country settings (18) (Supplementary Table S1). Two studies looked at low-income countries. Of the studies including multiple countries, these covered a range of low- middle, and high-income countries, although data were only included for LMICs from these studies (15–17, 19). Most studies (*n* = 12) were conducted at the sub-national level, focusing on specific regions within countries (Supplementary Table S1).

#### Study design and data sources

In terms of methodological design, four studies were qualitative, 14 were quantitative and five utilized mixed methods. The majority of qualitative studies relied on voluntary surveys, with some employing key informant interviews and focus group discussions. Many studies collected data through open access surveys conducted via the internet or social media, whereas others conducted interviews with identified individuals who were free to decline or accept to participate in the survey. Online surveys were more commonly administered in middle-income urban areas, whereas interviews and physical questioning were more often conducted in rural settings, notably in lower- or middle-lower income settings. In cases where administrative data were used for the analysis, sources included national health or administrative databases.

#### Study period

The majority of studies were conducted during or immediately following the COVID-19 pandemic. Where the research period was provided, this commonly extended from mid-2020 to late 2021. However, study duration varied substantially, ranging from a few days to several months, with an average of approximately one month.

#### Population group

Most studies focused on healthcare workers (*n* = 7) or parents, caregivers of children and pregnant women (*n* = 15), typically looking at routine or childhood immunizations. Of the seven studies that targeted healthcare workers, groups included family physicians, pediatricians, immunization program officials, vaccinators and other general healthcare professionals, across rural and urban settings. Among the 15 studies that included a focus on parents and caregivers, these included parents of infants or children of different age ranges and pregnant women (Supplementary Table S1). Three studies were conducted in the general population (19–21), and one examined vaccine uptake for the annual influenza vaccine conducted among adult university students in Lebanon (22).

#### Vaccines

Twelve studies evaluated the impact of the COVID-19 pandemic on routine childhood immunization in general. Eight studies focused on the impact of the pandemic on specific immunizations: one each on measles, meningitis, oral cholera, malaria, and influenza vaccines and three focused specifically on human papillomavirus (HPV) vaccines. Two studies had a combined focus, one on childhood vaccines and HPV, and one on adult and childhood routine vaccinations. One study evaluated vaccines in general.

#### Sample sizes

Study sample sizes varied widely depending on the research design, methodologies, and settings, ranging from 9 to over 630,000 participants (Supplementary Table S1).

### Quality assessment

MMAT scores from the quality assessment are presented in Supplementary Table S1. Most studies received a rating of five or four stars (*n* = 10 and *n* = 8, respectively), while three studies received a rating of three stars and one study received a rating of two stars. One study, Essoh et al. (23) 2022 received a rating of zero stars. All studies were included in the review regardless of their MMAT score.

### Impact of the COVID-19 pandemic on vaccine acceptance: BeSD analysis

Across the 23 studies reporting the impact of the COVID-19 pandemic on vaccine acceptance, data were categorized by BeSD category (Table 2), to understand how the pandemic impacted each BeSD variable. Findings belonging to the “Thinking and Feeling” category were the most commonly reported in 91% (*n* = 21/23) of studies. Over half (61%) of studies reported findings relating to the ‘Motivation' construct, while 43% of studies reported outcomes related to ‘Practical Issues' and ‘Social Processes'.

**Table 2:** Proportion of studies reporting each BeSD construct.

**BeSD categories**	**Number of studies (*N* = 23)**	**Percentage**
Thinking and feeling	21	91%
Motivation	14	61%
Practical issues	10	43%
Social processes	10	43%

Table 3 shows the number of studies reporting qualitative and quantitative outcomes for each BeSD sub-construct. The most commonly reported data were on “perceived disease risk” and “vaccine confidence”, indicating that the pandemic most commonly affected these aspects of people's perceptions of vaccination. Findings by BeSD category are described further in the following subsections.

**Table 3:** Number of studies reporting qualitative and quantitative outcomes for each sub-construct.

**BeSD sub-construct**	Qualitative^*^	Quantitative^*^
**Thinking and feeling**		
Perceived disease risk	10	8
Vaccine confidence	10	12
**Motivation**		
Intention to get recommended vaccines	5	11
**Practical issues**		
Availability	6	3
Affordability	2	0
Ease of access	4	3
Service quality	2	1
Respect from health worker	1	0
**Social process**		
Social norm	2	3
Health worker recommendation	2	5
Gender equity	0	1
**Other factors**		
Other	6	3

^*^Denotes number of outcomes rather than of studies overall.

#### Thinking and Feeling

Across studies, the COVID-19 pandemic was most commonly reported to affect “Thinking and Feeling” constructs around vaccination. Eighteen outcomes related to “perceived disease risk” were reported (10 qualitative and eight quantitative outcomes; Table 3). Several studies noted participant fear of contracting or spreading COVID-19 at health centers as one of the reasons for not seeking vaccination for themselves and their children during the pandemic (19, 23–28). In a study of HPV vaccine uptake in Kenya, parents reported that their daughters were reluctant to come to healthcare facilities due to fear of contracting COVID-19 (24). For some, this concern extended to the perceived risk of being infected by health workers (23). Perceptions were informed by a mix of genuine concerns, as well as the influence of rumors and misinformation resulting from the pandemic. Decouttere et al. (25) 2021 noted that fear of infection had contributed to a dip in confidence in the overall health system.

Similarly, studies reported a total of 22 outcomes related to the pandemic's impact on vaccine confidence, comprising 10 qualitative and 12 quantitative outcomes. Studies reported that information gaps resulted in confusion between perceptions of COVID-19 and the actual vaccines being administered (21, 23, 26, 29). For example, one study of parents/caregivers and age-eligible girls in Colombia reported some confusion between aspects of the prevention, transmission and side effects of COVID-19 and HPV vaccines, particularly among younger girls (29). Meanwhile, a study of vaccinators in Cameroon found that the COVID-19 pandemic was given as a key reason for community hesitancy toward HPV vaccination, alongside fear of infertility and the negative influence of social media (27). Specifically, vaccinators felt that the timing of HPV vaccine introduction during the COVID-19 pandemic was not ideal, and that the community had been insufficiently sensitized to the HPV vaccine leading to confusion with COVID-19 (27). Some vaccinators reported that the community believed the HPV vaccine was a cover for the pharmaceutical industries and government to infect them with COVID-19 (27). Fear of being intentionally or unintentionally administered the COVID-19 vaccine played a role in reducing vaccination-seeking behavior in several studies (21, 30, 31). One large-scale study in sub-Saharan Africa found that general vaccine confidence had declined in all eight study countries from 2020 to 2022 (15). Reductions in vaccine confidence rates ranged from 3% in South Africa to 13% in Uganda (15).

However, some studies noted a positive impact of the COVID-19 pandemic on people's thinking and feeling around vaccination. Cordoba-Sanchez et al. noted that discussions around COVID-19 vaccines helped to de-mystify anti-vaccine sentiments for HPV vaccines leading to greater understanding of the vaccine and increased acceptance (29). Another study showed that parental attitudes toward childhood vaccination had remained mostly positive throughout the pandemic, with 85.7% of parents (*n* = 377/440) stating that they thought positively about childhood vaccines (32).

#### Motivations

Fourteen studies highlighted factors related to changes in motivations around vaccination for adult and childhood vaccinations, across 16 outcomes relating to “Intention to get vaccinated”. Six of the studies focused on vaccine hesitancy, albeit with varying measurement scales (16, 20, 32–35).

Several studies were conducted in Türkiye with varying results. In two of the studies, parents' perceptions either remained the same or were largely positive, while one study showed that vaccine hesitancy had increased. One study examined differences in parental attitudes toward childhood vaccination during two peak times of the pandemic, August 2020 and February 2021 (34). It found significantly lower levels of hesitancy toward childhood vaccination among parents who had experienced COVID-19 either directly or indirectly than those who had not (with mean WHO 10-item Vaccine Hesitancy Scale scores of 20.0 vs. 24.7%, respectively, *p* < 0.001) (34). The study also found a statistically significant increase (*p* = 0.008) in parental hesitancy toward childhood vaccines from 10.6% of the parents surveyed in 2020 to 20% of parents surveyed in 2021 (34). A second study found that 6.4% of parents remained hesitant about childhood vaccination before and after the pandemic, while 5% had become less hesitant about the importance of vaccines during the pandemic (32). Another study conducted in Türkiye aimed to understand the vaccination opinions of pregnant women and the effect of the COVID-19 pandemic (35). The study found that hesitancy mostly stayed the same, with 63.9% of pregnant women reporting that the COVID-19 pandemic had not changed their opinions on vaccination, while 28.9% reported that their hesitation about vaccination had lessened due to the pandemic. Half of the respondents recorded no changes in future intent to get vaccinated (50.6%), while 44.1% reported that the pandemic had a positive effect on intention to vaccinate. In addition, 50% of women reported that the COVID-19 pandemic had a positive effect on intention to have their children vaccinated in the future, while 41.4% reported no change in future intent to vaccinate their children (35). Finally, when asked about reasons for vaccination during the pandemic in another study from Türkiye, 43.9% of parents/caregivers reported that pediatric vaccines are vital and must be administered (36).

In addition, a study in India reported a significant increase in the percentage of mothers expressing vaccine hesitancy, from 5.3% pre-pandemic to 38% during the pandemic (*p* = 0.003) (33). Furthermore, a study in the Eastern Mediterranean region identified several factors that affected parental attitudes toward childhood vaccination. Among LMICs, mother's age, residence (urban, rural), country income level, gender of the child, total number of children, and source of information regarding vaccines (social media and internet) had a direct effect on parental attitudes toward childhood vaccination, with an effect size of 2.85 in lower-middle-income countries and 7.17 in upper middle-income countries (16). However, a study in Jordan found that a high proportion of parents/caregivers had positive beliefs toward childhood vaccination during the pandemic, with 93.1% of parents reporting that adherence to the child vaccination schedule is essential (28).

A study of the pandemic's influence on parents' attitudes and behaviors toward meningococcal vaccination found that parents/caregivers in Brazil (mean score: 3.4; SD: 1.0), and Argentina (mean score: 3.5; SD: 0.8) would be more likely to have their children catch up on missed or delayed vaccinations than parents in the high-income study countries (except Italy) (17).

#### Practical issues

Across the 10 studies reporting “Practical Issues” relating to vaccine acceptance, “availability” was the most evoked construct, with nine outcomes reported (six qualitative and three quantitative). The second most common construct was “ease of access” with seven outcomes reported. Three outcomes were reported related to “service quality”, two outcomes related to “affordability” issues, and one outcome related to “respect from health worker”.

Several studies reported that the pandemic affected the availability and accessibility of immunization services, including home visits by vaccinators (19, 21, 24, 28, 29, 31, 32). One study investigated the contribution of vaccine hesitancy to under-immunization and a measles outbreak in Rwanda (25). The study found that the reduction of childhood vaccination sessions during the pandemic made it less convenient for caregivers to seek vaccination and increased complacency in vaccination-seeking behavior due to limited contact between health workers and caregivers (25).

In Nigeria, a study on the impact of the pandemic on vaccination services found a lack of proper training for healthcare workers in managing the pandemic, which may have contributed to hesitancy among caregivers, discouraging them from seeking other health services (23). Meanwhile, a study in Kenya noted that stock-out of vaccines worsened during the pandemic (24). In the same study, caregivers also reported the attitudes and behaviors of healthcare workers as a barrier to their willingness to return for future vaccines (24). Additionally, a study in Colombia noted that the COVID-19 pandemic further limited access to HPV vaccines and reduced the importance of HPV-related health campaigns (29). A further study from Kenya found that the COVID-19 pandemic and the government's response strategies negatively affected HPV vaccine uptake, as key venues for HPV advocacy, such as schools and churches, were closed (24). However, one study in Northern Ghana reported that delays in childhood vaccinations during the COVID-19 pandemic were not attributed to a lack of vaccines or healthcare staff (26).

#### Social processes

Ten studies reported outcomes related to “Social Processes” under three constructs: social norms (5 outcomes), health worker recommendations (seven outcomes), and gender equity (one outcome). One study of 26 upper- and middle-income countries reported the impact of the COVID-19 pandemic on social factors affecting missed or delayed vaccination. Of the nine middle-income countries included in the study, respondents evoked saving immunization services for those most in need (*n* = 172, 11%) and recommendation to delay vaccination by health provider (*n* = 137, 8%) as the reasons for missed or delayed vaccination during the COVID-19 pandemic (19).

Several other studies noted religious reasons affecting decisions to vaccinate (21, 24, 31). In one study of the COVID-19 pandemic's impact on routine childhood vaccination in Nigeria, religious leaders were noted to encourage childhood vaccination (31). In another study, religious beliefs were identified as a factor driving low demand or vaccine hesitancy for routine childhood immunizations (24). One study examining an oral cholera mass vaccination campaign in Cameroon during the COVID-19 pandemic reported factors related to gender equity (21). Among those who refused vaccination, 8% of women said that they were not decision-makers regarding whether to get vaccinated (21).

#### Other factors

Nine studies reported ‘Other' factors encapsulated by elements not specific to the BeSD benchmarks. One study found that 67.6% of the reasons for mothers delaying routine pediatric vaccination during the COVID-19 pandemic related to waiting until the number of COVID-19 cases decreased out of fear of contracting the virus (36). In a univariate analysis, anxiety-fear had a statistically significant effect on delayed vaccination status (*p* = 0.001) (36). In another study of childhood vaccine uptake in Jordan, 57% of those who had delayed their child's vaccines admitted that the delay was related to the COVID-19 pandemic (28). A study in Northern Ghana reported misinformation from the general public as another factor accounting for the decline in childhood immunizations (26). Further, a study of 26 countries identified that more households in middle-income countries reported missed childhood vaccinations compared with high-income countries during the COVID-19 pandemic (7.6 vs. 3.0%, respectively) (19).

#### Overall impact of the COVID-19 pandemic on vaccine acceptance

**Figure 3:**
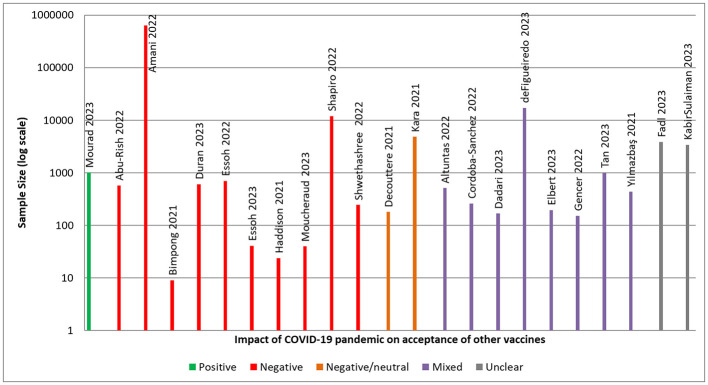
Overall direction of study findings on vaccine acceptance. Positive: increase in measures of vaccine acceptance; negative: decrease in measures of acceptance; mixed: if both positive and negative effects were reported; neutral: no overall change reported or not measured; unclear: results were ambiguous or insufficient to support classification.

Figure 3 shows the overall direction of the reported impact of the COVID-19 pandemic on vaccine acceptance attitudes, intentions, and actions in each study. One study found a positive effect of the pandemic on vaccine acceptance, while 12 reported a negative or negative/neutral effect. Eight studies reported that the COVID-19 pandemic had both positive and negative effects on vaccine acceptance, while the direction of effect was unclear in two studies.

## Discussion

This systematic review provides novel insights into the impact of the COVID-19 pandemic on general vaccine acceptance in LMICs directly following the pandemic, through the lens of the underlying behavioral and social drivers of vaccination. The analysis suggests that the COVID-19 pandemic affected vaccine acceptance in complex, context-dependent ways, impacting people's thinking and feeling, motivations, practical issues, and social processes around vaccination. Overall, studies most commonly reported that the COVID-19 pandemic had a negative or negative/neutral effect on vaccine acceptance attitudes, intentions, and actions. Across studies, the COVID-19 pandemic was most frequently found to affect people's thinking and feeling around vaccination. Specifically, perceived disease risk and vaccine confidence had the most impact on peoples' decisions to seek childhood and/or adult vaccinations during and after the COVID-19 pandemic. Fear of contracting COVID-19 was a common reason reported for not seeking vaccination during the pandemic, indicating that risk perception has the potential to contribute to the increase or decline of vaccine confidence. Vaccine confidence was also prominently evoked in the studies, although studies reported positive, neutral, and negative effects of the pandemic on vaccine confidence.

In terms of motivation to vaccinate, studies reported both increases and decreases in vaccine hesitancy and intention to vaccinate due to the pandemic, indicating that the impact varied by the particular population and context. Interestingly, one study found significantly lower levels of hesitancy toward childhood vaccination among parents who had directly or indirectly experienced COVID-19, suggesting that personal experiences can modify vaccine acceptance and hesitancy (34). Ten studies reported the impact of the COVID-19 pandemic on social processes related to vaccination, under three constructs: social norms, health worker recommendations and gender equity. Studies reported both positive and negative effects of religious beliefs on vaccination. Among the practical factors impacted by the pandemic across studies, issues related to “vaccine availability” and “ease of access” were the most commonly reported. These practical factors directly impact vaccine acceptance, as they represent barriers that can either facilitate or hinder individuals' ability and motivation to seek vaccination. Only one study addressed gender equity, finding that 8% of people refusing vaccination were women who said they were not decision-makers for vaccination (21). Although evidence from this review was limited, the finding reflects research showing that women in LMICs often lack decision-making authority around vaccination (37).

In context, the findings add to the body of literature on the complex factors affecting vaccine hesitancy, confidence, and acceptance. In agreement with the literature, the analysis demonstrates how perceptions of vaccination, disease risk and vaccine confidence can be affected differently, depending on the context and population. In terms of risk perception, evidence has shown that people who do not experience negative consequences for not immunizing are less likely to pursue vaccination in the future (38). Previous vaccination status is also an indicator for future vaccination. Research has shown that people who had received an influenza vaccine were more likely to get it again the following year (39), and that those with a history of influenza vaccination were also more likely to receive the COVID-19 vaccine compared with those who had not received influenza vaccination (40).

Although the review identified perceived risk as a key factor affecting vaccine acceptance, studies did not clarify whether individuals' perceptions of their personal susceptibility to infection and the perceived severity of a disease influenced vaccination uptake together or as two separate factors. A meta-analysis of the relationship between risk perception and health behaviors suggests that personal susceptibility and risk severity influence behaviors distinctively (41). The finding by Decouttere et al. (25) that the fear of infection contributed to a decline in confidence in the health system adds another layer of complexity to the relationship between perceived risk and confidence. Growing evidence of the long-term burden of post-COVID conditions, such as fatigue, cognitive issues, and persistent exertional dyspnea, may also shape risk perceptions and demand for vaccination beyond the initial pandemic period (42–44).

In this review, studies also found that rumors and a lack of accurate information negatively impacted perceptions of vaccination during the COVID-19 pandemic. Information gaps and misinformation are known to weaken adherence to public health measures (45), reduce willingness to get vaccinated, and decrease the likelihood of recommending vaccination to others (46). Although rumors and misinformation negatively impacted vaccination uptake before COVID-19, the pandemic led to a dramatic uptick in vaccine- and health-related misinformation. Digital and social media have also exacerbated the situation, making it possible for misinformation to spread much faster and wider than in the pre-digital age (47, 48). This review highlights how the presence or absence of accurate information can either positively or negatively impact vaccine confidence. A study from Colombia exploring the factors influencing HPV-vaccine hesitancy reported that COVID-19 discussions helped demystify HPV-related concerns and rumors (29). In addition, the study identified that receiving positive information about the HPV vaccine was significantly associated with the likelihood of getting vaccinated (29). This is supported by a previous systematic review of the barriers and facilitators of HPV vaccination in sub-Saharan Africa, which reported insufficient knowledge of HPV to be a major barrier to vaccine uptake in six out of 20 studies (49). Misinformation was also reported as an obstacle to vaccination in five of the studies (49). Like COVID-19 vaccines, HPV vaccines have been substantially affected by rumors and misinformation regarding their safety and efficacy (49).

Gaps in health worker knowledge were found to negatively influence vaccination acceptance in some of the studies identified in this review. Evidence showed that health worker information gaps around the science and development of vaccines precluded them from providing vital information to patients for the HPV vaccine in Armenia (50). The literature points to the importance of strengthening the knowledge base of health providers to improve vaccine confidence and acceptance (51, 52). Interventions that have been shown to mitigate vaccine hesitancy include strengthening the interpersonal communication capacities of health workers and targeted trainings on effective approaches to improve vaccine acceptance and uptake (51, 53, 54).

In general, the pandemic had varying effects on vaccine acceptance across different subgroups, for example, with studies reporting both positive, negative, and mixed effects on vaccine acceptance across parents and caregivers. However, the four studies on HPV vaccines found that the COVID-19 pandemic caused fear and/or confusion about HPV vaccination among the community, parents, caregivers, and girls eligible for vaccination. Studies reported confusion over aspects of COVID-19 and HPV vaccination, particularly among younger girls. People also shared concerns that receiving the HPV vaccine would lead to them being unintentionally vaccinated against COVID-19 or intentionally infected with COVID-19. The findings suggest that the pandemic may have amplified doubts and fears for newer vaccines, such as the HPV vaccine, with which people have less familiarity. However, as highlighted in the aforementioned study by Cordoba-Sanchez et al., discussion of COVID-19 vaccines may also increase awareness and acceptance of newer vaccines, like the HPV vaccine, by providing greater understanding of vaccination in general (29).

Importantly, this study adds to growing evidence of global volatility in vaccine acceptance and confidence, including in high-income settings. Other systematic reviews and observational studies on vaccine hesitancy reveal similar relationships to those identified in this review, showing how factors like risk perception, trust, past vaccination behavior and socio-structural constraints interact to influence vaccine decisions (55, 56). For example, a systematic review of COVID-19 vaccine hesitancy among Italian parents found that factors such as risk perception, trust in healthcare workers, the availability of scientific information, and prior vaccination influenced parents' decision to vaccinate their children (55). Similarly, a systematic review of the COVID-19 pandemic's impact on influenza vaccine uptake among European healthcare workers found that prior vaccination was a key factor for vaccination compliance (57). Specifically, healthcare workers who had been vaccinated in previous influenza seasons and were willing to receive or had received the COVID-19 vaccine were more likely to get the influenza vaccine (57). LMICs face additional challenges to vaccine acceptance and uptake, as a result of limited access to healthcare, resource constraints, and competing health priorities. However, even when vaccines are readily available, studies have identified persistent immunity gaps, highlighting how complex and deep-seated behavioral and structural factors affect vaccine acceptance (58, 59).

Summarizing the impact of the COVID-19 pandemic on vaccination acceptance in LMICs remains a complex task due to the wide array of outcomes highlighted in the studies. The review did not evaluate associations across the different BeSD benchmarks, although it recognizes that people may experience counteracting factors throughout their vaccination journey that ultimately inform vaccination behaviors. In addition, the studies highlight the context-specific nature of the factors affecting vaccine acceptance, which limits the generalizability of specific insights. Studies were also conducted during different phases of the COVID-19 pandemic, which could influence perceptions and views, as these may have differed over the course of the pandemic. Future systematic research to stratify the impact of the pandemic by phase (e.g., early pandemic, vaccine rollout, subsequent waves) could offer a more nuanced understanding of how different phases of a pandemic influence vaccine acceptance and uptake. In addition, as the study intended to look at the pandemic and immediate post-pandemic period, up to November 2023, the impact of the pandemic on vaccine acceptance in more recent years was not assessed. Future research to evaluate the broader impact of the pandemic over a longer time frame would be valuable to investigate the lasting impacts of changes in vaccine acceptance. Further, this study did not assess the impact of the pandemic on vaccine acceptance in high-income countries, and future work to characterize and contrast the impact in high-income settings would provide a richer understanding of how pandemics affect vaccine acceptance.

In addition, while only one of the papers discussed the impact of the COVID-19 pandemic on equity-related factors, equity plays an important role in vaccine acceptance. Literature suggests that intersecting determinants such as gender may exert a varying impact, potentially introducing confounding factors across different demographics (5). Consequently, it would be valuable to further examine the impact of equity, including gender-related factors on vaccine acceptance in future research. Specifically, future research should systematically explore the intersection of gender, socio-economic status, urban-rural residence and marginalization through qualitative and quantitative methods. Such analyses would provide a better understanding of how equity-related factors affect vaccine acceptance and decision-making. Nevertheless, this review provides insights into the impact of the COVID-19 pandemic on vaccine hesitancy and acceptance, demonstrating its complex effects on people's thoughts, feelings, motivations, and actions around vaccination. The findings also underscore the volatility and variability of vaccine confidence, as something that can be gained or lost.

## Conclusions

This systematic review provides the first comprehensive assessment of the COVID-19 pandemic's impact on general vaccine acceptance during the pandemic and in the immediate post-pandemic period, characterized by the BeSD framework. The findings demonstrate that the COVID-19 pandemic affected people's perceptions, decisions, and actions around vaccination in a myriad of ways. Most studies (91%) reported outcomes related to the “Thinking and Feeling” BeSD category, 61% reported findings relating to the ‘Motivation' construct, and 43% reported outcomes related to ‘Practical Issues' and ‘Social Processes'. Studies described both increases and decreases in vaccine hesitancy and intention to vaccinate due to the pandemic. However, studies most commonly reported that the COVID-19 pandemic had a negative or neutral effect on vaccine acceptance attitudes, intentions, and actions.

Overall, the review highlights the importance of understanding the specific factors affecting people's decision to vaccinate, so tailored and targeted strategies can be developed. To this end, validated frameworks and models, such as BeSD, should be leveraged to design social and behaviorally informed strategies to improve vaccination uptake. The insights from this study can be used to guide vaccination catch-up efforts and address the social, behavioral, and practical barriers to vaccination that were exacerbated by the COVID-19 pandemic.

## Data Availability

The original contributions presented in the study are included in the article/Supplementary material, further inquiries can be directed to the corresponding author.
