# Supplementary material for: The impact of COVID-19 on general vaccine acceptance in low- and middle-income countries: a systematic review
Source: Front Public Health. 2026 Feb 23;14:1764389. doi: 10.3389/fpubh.2026.1764389 (PMC12968277; doi:10.3389/fpubh.2026.1764389)
Supplement: Supplementary file 1 [file Table_1.docx]

Supplementary Material

# Supplementary Figures and Tables

**Table S1. Study Characteristics and MMAT Rating**

| **Author** | **Study design** | **Geographical scope** | **Country(s)** | **Country income** | **Population** | **Sample size (n)** | **Vaccine** | **BeSD/SBC construct** | **MMAT scoring** |
| --- | --- | --- | --- | --- | --- | --- | --- | --- | --- |
| Abu-Rish (2022) (1) | Quantitative | National Level | Jordan | Low middle income | Adults aged ≥18 who were Jordanian parents/caregivers for vaccine-eligible children (0–23 months) | 568 | Routine childhood vaccines | Thinking and Feeling; Motivation Practical Issues; Social Processes; Other: other outcome measure denoted by "vaccine delay" | *** |
| Altuntas (2022) (2) | Quantitative | Sub-national Level | Türkiye | Upper middle income | Parents of children aged 0–24 months | 513 | Routine childhood vaccines | Thinking and Feeling; Motivation; Social Processes; Other: Other denotes 'delay in vaccination' | *** |
| Amani (2022) (3) | Quantitative | Sub-national Level | Cameroon | Low middle income | General population aged >12 months including pregnant women | 630,109 | Oral cholera vaccines | Thinking and Feeling; Social Processes; Practical Issues | *** |
| Bimpong (2021) (4) | Mixed methods study | Sub-national level | Ghana | Low middle income | Staff and caregivers at Tamale Teachers Training Hospital | 9 | Routine childhood vaccines | Thinking and Feeling; Practical Issues; Other | **** |
| Cordoba-Sanchez (2022) (5) | Mixed methods study | Sub-national level | Colombia | Upper middle income | Parents and caregivers as well as HPV eligible girls | Quantitative: 196 Qualitative: 63 | HPV | Thinking and Feeling; Social Processes, Practical Issues; Other | **** |
| Dadari (2023) (6) | Mixed methods study | Sub-national level | Nigeria | Low middle income | 1.KII participants were immunization experts across government and partner agencies at the state and LGAs, with health facility managers 2.Frontline vaccinators and caregivers of  eligible infants from 2018 to 2021 were selected for the  FGD session | FGD: 129 KII: 40 | Routine childhood vaccines | Thinking and Feeling; Motivation; Practical Issues; Social Processes; Other | ** |
| Decouttere (2021) (7) | Qualitative | National level | Rwanda | Low income | Caregivers and health workers | Caregivers: 161 Vaccination service providers: 11 Health workers: 9 | Under immunization & Measles | Thinking and Feeling; Practical Issues | **** |
| de Figueiredo (2023) (8) | Quantitative | Multi-country |  | Other: Mix of low and low and middle income |  | 17,187 | General vaccines | Thinking and Feeling; Motivation | ***** |
| Duran (2023) (9) | Quantitative | Sub-national level | Türkiye | Upper middle income | Caregivers | 610 | Routine childhood vaccines | Thinking and Feeling; Motivation | ***** |
| Elbert (2023) (10) | Quantitative | Sub-national level | Indonesia | Upper middle income | Mothers of children aged 0–12 month | 196 | Routine childhood vaccines | Thinking and Feeling; Motivation | **** |
| Essoh (2022) (11) | Qualitative | National level | Nigeria | Low middle income | Caregivers and health care workers | Healthcare workers: 517 Caregivers: 180 | Routine childhood vaccines | Thinking and Feeling; Practical Issues; Social Processes | 0 stars |
| Essoh (2023) (12) | Qualitative | Sub-national level | Kenya | Low middle income | 1.Immunization officials 2. Caregivers of children 0–23 months and adolescent girls eligible for HPV vaccines | Immunization officials: 25 Caregivers: 16 | Routine childhood vaccines and HPV | Thinking and Feeling; Practical Issues; Social Processes; Other | ***** |
| Fadl (2023) (13) | Quantitative | Multi-country | Afghanistan, Egypt, Iraq, Jordan, Libya, Palestine, Pakistan, , Syria, Tunisia | Low and middle income | Parents of children aged 6 months to 18 years | 3873 | Routine childhood vaccines | Motivation | ***** |
| Gencer (2022) (14) | Quantitative | National | Türkiye | Upper middle income | Pregnant women | 152 | Routine childhood vaccines | Thinking and Feeling; Motivation | ***** |
| Haddison (2021) (15) | Mixed methods | Sub-national level | Cameroon | Low middle income | Vaccinators, health care workers | 24 | HPV | Thinking and Feeling; Other | ***** |
| Kabir Sulaiman (2023) (16) | Quantitative | National level | Nigeria | Low middle income | Nigerian adults | 3377 | Malaria | Motivation | **** |
| Kara (2021) (17) | Quantitative | Sub-national Level | Türkiye | Upper middle income | Family practitioners and pediatricians | Family practitioners: 2860 Pediatricians:1908 Pediatric specialists:70 | Routine childhood vaccines | Thinking and Feeling; Motivation; Social Processes | ***** |
| Moucheraud (2023) (18) | Qualitative | Sub-national level | Malawi | Low income | Caregivers (parents or guardians) of pre-adolescent girls in Malawi | 40 | HPV | Thinking and Feeling; Social Processes | **** |
| Mourad (2023) (19) | Quantitative | National level | Lebanon | Low middle income | University students | 1016 | Influenza vaccine | Thinking and Feeling; Motivation; Other | ***** |
| Shapiro (2022) (20) | Quantitative | Multi-country | Middle income: Brazil, China, India, Indonesia, Malysia, Mexico, Philippines, Thailand, Vietnam | Middle income | National panels of adults who periodically complete surveys online | 11,995 | Adult and routine childhood vaccines | Thinking and Feeling; Social Processes; Practical Issues; Other | ***** |
| Tan (2023) (21) | Mixed methods | Multi-country | Brazil, Argentina | Middle income | Parents of eligible children 0–4 years and adolescents 11–18 years | 1002 | Meningitis | Thinking and Feeling; Motivation | **** |
| Shwethashree   (2022) (22) | Quantitative | Sub-national level | India | Middle income | Mothers of children under 6 years | 246 | Routine childhood vaccines | Thinking and Feeling; Motivation; Practical Issues | **** |
| Yılmazbaş (2021) (23) | Quantitative | National level | Türkiye | Upper middle income | Parents of children in Türkiye | n = 440 | Routine childhood vaccines | Thinking and Feeling; Motivation | ***** |

FDG, focus group discussion; HPV, human papillomavirus; KII, key informant interview; LGA, local government area; UAE, United Arab Emirates.

Country income classifications reported as per the World Bank income classification for 2023 (24)

**References**

1. Abu-rish EY, Bustanji Y, Abusal K. Nationwide Routine Childhood Vaccination Coverage During the COVID-19 Pandemic in Jordan: Current Situation, Reasons, and Predictors of Vaccination. International Journal of Clinical Practice. 2022;2022(1):7918604.

2. Baktır Altuntaş S, Kara Elitok G. Routine Pediatric Vaccination During Pandemic: Attitudes of Parents. Turk Arch Pediatr. 2022;57(3):342-8.

3. Amani A, Ngo Bama S, Dia M, Nguefack Lekelem S, Linjouom A, Mossi Makembe H, et al. Challenges, best practices, and lessons learned from oral cholera mass vaccination campaign in urban Cameroon during the COVID-19 era. Vaccine. 2022;40(47):6873-9.

4. Bimpong KA, Nuertey BD, Seidu AS, Ajinkpang S, Abdul-Mumin A. Decline in Uptake of Childhood Vaccinations in a Tertiary Hospital in Northern Ghana during the COVID-19 Pandemic. Biomed Res Int. 2021;2021:6995096.

5. Cordoba-Sanchez V, Lemos M, Tamayo-Lopera DA, Sheinfeld Gorin S. HPV-Vaccine Hesitancy in Colombia: A Mixed-Methods Study. Vaccines (Basel). 2022;10(8).

6. Dadari I, Sharkey A, Hoare I, Izurieta R. Analysis of the impact of COVID-19 pandemic and response on routine childhood vaccination coverage and equity in Northern Nigeria: a mixed methods study. BMJ Open. 2023;13(10):e076154.

7. Decouttere C, Banzimana S, Davidsen P, Van Riet C, Vandermeulen C, Mason E, et al. Insights into vaccine hesitancy from systems thinking, Rwanda. Bull World Health Organ. 2021;99(11):783-94d.

8. de Figueiredo A, Temfack E, Tajudeen R, Larson HJ. Declining trends in vaccine confidence across sub-Saharan Africa: A large-scale cross-sectional modeling study. Hum Vaccin Immunother. 2023;19(1):2213117.

9. Duran S, Duran R, Acunaş B, Şahin EM. Changes in parents' attitudes towards childhood vaccines during COVID-19 pandemic. Pediatr Int. 2023;65(1):e15520.

10. Elbert B, Zainumi CM, Pujiastuti RAD, Yaznil MR, Yanni GN, Alona I, et al. Mothers' knowledge, attitude, and behavior regarding child immunization, and the association with child immunization status in Medan City during the COVID-19 pandemic. IJID Reg. 2023;8(Suppl):S22-6.

11. Essoh TA, Adeyanju GC, Adamu AA, Ahawo AK, Aka D, Tall H, et al. Early Impact of SARS-CoV-2 Pandemic on Immunization Services in Nigeria. Vaccines (Basel). 2022;10(7).

12. Essoh TA, Adeyanju GC, Adamu AA, Tall H, Aplogan A, Tabu C. Exploring the factors contributing to low vaccination uptake for nationally recommended routine childhood and adolescent vaccines in Kenya. BMC Public Health. 2023;23(1):912.

13. Fadl N, Al Awaidy ST, Elshabrawy A, Makhlouf M, Ibrahim SA, Abdel-Rahman S, et al. Determinants of parental seasonal influenza vaccine hesitancy in the Eastern Mediterranean region: A cross-sectional study. Front Public Health. 2023;11:1132798.

14. Gencer H, Özkan S, Vardar O, Serçekuş P. The effects of the COVID 19 pandemic on vaccine decisions in pregnant women. Women Birth. 2022;35(3):317-23.

15. Haddison E, Tambasho A, Kouamen G, Ngwafor R. Vaccinators' Perception of HPV Vaccination in the Saa Health District of Cameroon. Front Public Health. 2021;9:748910.

16. Kabir Sulaiman S, Isma'il Tsiga-Ahmed F, Sale Musa M, Kabir Sulaiman A, Muhammad Dayyab F, Ab Khan M, et al. Prevalence, determinants, and reasons for malaria vaccine hesitancy among caregivers of under-five children in Nigeria: Results from a nationwide cross-sectional survey. Vaccine. 2023;41(8):1503-12.

17. Kara A, İlbay S, Topaç O, Arabulan EA, Tezer H, Tavukçu N, et al. Alteration in vaccination rates and an evaluation of physicians' perceptions of the possible impact of the SARS-CoV-2 pandemic on childhood vaccinations in Ankara, Turkey. Hum Vaccin Immunother. 2021;17(10):3457-62.

18. Moucheraud C, Whitehead HS, Songo J, Szilagyi PG, Hoffman RM, Kaunda-Khangamwa BN. Malawian caregivers' experiences with HPV vaccination for preadolescent girls: A qualitative study. Vaccine X. 2023;14:100315.

19. Mourad N, Mourad L, Hammoudi Halat D, Farah Z, Hendaus M, El Sayed Trad I, et al. Factors Affecting Influenza Vaccination Uptake and Attitudes among Lebanese University Students: The Impact of Vaccination Promotional Programs and COVID-19 Pandemic. Vaccines (Basel). 2023;11(5).

20. Shapiro GK, Gottfredson N, Leask J, Wiley K, Ganter-Restrepo FE, Jones SP, et al. COVID-19 and missed or delayed vaccination in 26 middle- and high-income countries: An observational survey. Vaccine. 2022;40(6):945-52.

21. Tan LLJ, Safadi MAP, Horn M, Regojo Balboa C, Moya E, Schanbaum J, et al. Pandemic's influence on parents' attitudes and behaviors toward meningococcal vaccination. Hum Vaccin Immunother. 2023;19(1):2179840.

22. Shwethashree M, Vanmathi A, Saurish, Amoghashree, Narayanamurthy MR, Gopi A. Did this pandemic trigger a spike in mothers' hesitancy over their children's routine immunizations? -A cross sectional study. Clin Epidemiol Glob Health. 2022;15:101023.

23. Yılmazbaş NP, Terzi Ö, Özçeker D. Did COVID-19 Pandemic Changed Parents’ Approach to Vaccination? Journal of Clinical Practice and Research (Formerly Erciyes Medical Journal). 2021;43(2):130-4.

24. The World Bank. World Bank Country and Lending Groups - 2023 2023 [Available from: <https://datahelpdesk.worldbank.org/knowledgebase/articles/906519-world-bank-country-and-lending-groups>.
