# Supplementary material for: The impact of COVID-19 on general vaccine acceptance in low- and middle-income countries: a systematic review
Source: Front Public Health. 2026 Feb 23;14:1764389. doi: 10.3389/fpubh.2026.1764389 (PMC12968277; doi:10.3389/fpubh.2026.1764389)
Supplement: Supplementary file 2 [file Data_Sheet_1.docx]

Supplementary Material

S1. Search Strategy

(Vaccination Hesitancy/ OR Immunization Programs/ OR Immunization Program OR Vaccination Campaign OR Vaccination Campaigns OR Vaccination Promotion OR Vaccination Promotions OR Vaccination Awareness OR Vaccination Hesitancies OR Vaccine Hesitancy OR Vaccine Delay OR Vaccine Delays OR Vaccination Delay OR Vaccination Delays OR Vaccine confidence OR Vaccine acceptance OR Vaccine hesitant OR Vaccine-hesitant OR Vaccin* adj3 (Campaign* OR Promot* OR Aware* OR Hesitan* OR Delay* OR confidence OR accept* OR Vaccine hesitant) OR Immuni?ation Program*) AND (COVID-19/ OR COVID-19 OR SARS-CoV-2) AND (Developing Countries/OR Developing Country OR Developing Nations OR Developing Nation OR Least Developed Countries OR Least Developed Country OR Less-Developed Countries OR Less Developed Countries OR LMICs OR Third-World Countries OR Third World Countries OR Third-World Country OR Third-World Nations OR Third World Nations OR Third-World Nation OR Under-Developed Countries OR Under Developed Countries OR Under-Developed Country OR Under-Developed Nations OR Under Developed Nations OR Low and Middle Income Countries OR Less-Developed Nations OR Less Developed Nations OR Less-Developed Nation OR Low Income Countries OR Low Income Country OR Middle Income Countries OR Middle Income Country OR Lower-Middle-Income Country OR Lower Middle Income Country OR Lower-Middle-Income Countries OR  Latin America/ OR  Middle East/ OR Africa South of the Sahara/ OR Pacific Islands/ OR emerging economies OR afghan* OR africa* OR albania* OR algeria* OR angola* OR antigua* OR barbuda* OR argentin* OR armenia* OR aruba* OR azerbaijan* OR bahrain* OR bangladesh* OR bengal* OR bangal* OR barbados* OR barbadian* OR bajan OR bajans OR belarus* OR belorus* OR byelarus* OR byelorus* OR belize* OR benin* OR dahomey OR bhutan* OR bolivia* OR bosnia* OR herzegovin* OR botswan* OR batswan* OR bechuanaland* OR brazil* OR brasil* OR bulgaria* OR burkina* OR burkinese* OR upper volta* OR burundi* OR urundi* OR cabo verde* OR cape verde* OR cambodia* OR kampuchea* OR khmer* OR cameroon* OR cameroun* OR ubangi shari* OR chad* OR chile* OR china* OR chinese OR colombia* OR comoro* OR comore* OR comorian* OR mayotte* OR congo* OR zaire* OR costa rica* OR "cote d'ivoir*" OR "cote d' ivoir*" OR cote divoir* OR cote d ivoir* OR ivory coast* OR ivorian* OR croatia* OR cuba OR cuban OR cubans OR "cuba's" OR cyprus* OR cypriot* OR czech* OR djibouti* somaliland* OR dominica* OR ecuador* OR egypt* OR united arab republic* OR el salvador* OR salvadoran* OR guinea* OR equatoguinea* OR eritrea* OR estonia* OR eswatini* OR swaziland* OR swazi* OR swati* OR ethiopia* OR fiji* OR gabon* OR gabonese* OR gabonaise* OR gambia* OR ((georgia OR georgian OR georgians) not (atlanta OR california OR florida)) OR ghana* OR gibraltar* OR greece* OR greek* OR grecian* OR grenada* OR grenadian* OR guam* OR guatemala* OR guyana* OR guiana* OR guyanese* OR haiti* OR hispaniola* OR hondura* OR hungary* OR hungarian* OR india* OR indonesia* OR iran* OR iraq* OR isle of man* OR jamaica* OR jordan* OR kazakh* OR kenya* OR karabati* OR korea* OR kosovo* OR kosova* OR kyrgyz* OR kirgiz* OR kirghiz* OR laos OR lao OR laotian* OR latvia* OR lebanon* OR lebanese* OR lesotho* OR lesothan* OR lesothonian* OR basutoland* OR mosotho* OR basotho* OR liberia* OR libya* OR jamahiriya* OR lithuania* OR macedonia* OR madagasca* OR malagasy* OR malawi* OR nyasaland* OR malaysia* OR malay* federation OR maldives* OR maldivian* OR indian ocean OR mali OR malian* OR "mali's" OR malta OR maltese* OR "malta's" OR micronesia* OR marshallese* OR kiribati* OR marshall island* OR nauru OR nauran OR nauruans OR "naurian's" OR mariana OR marianas OR palau OR paluan* OR tuvalu* OR mauritania* OR mauritan* OR mauritius* OR mexico* OR mexican* OR moldova* OR moldovia* OR mongol* OR montenegr* OR morocco* OR moroccan* OR ifni OR mozambique* OR mozambican* OR myanmar* OR burma* OR burmese OR namibia* OR nepal* OR new caledonia* OR netherlands antill* OR nicaragua* OR niger* OR oman OR omani OR omanis OR "oman's" OR pakistan* OR palestin* OR gaza* OR west bank* OR panama* OR paraguay* OR peru OR peruvian* OR "peru's" OR philippine* OR philipine* OR phillipine* OR phillippine* OR filipino* OR filipina* OR poland* OR polish OR pole OR poles OR portugal* OR portuguese OR puerto ric* OR romania* OR russia* OR ussr* OR soviet* OR rwanda* OR rwandese OR ruanda* OR ruandese OR samoa* OR navigator island* OR pacific island* OR polynesia* OR sao tome* OR santomean* OR saudi arabia* OR saudi OR saudis OR senegal* OR serbia* OR seychell* OR sierra leone* OR slovak* OR sloven* OR melanesia* OR solomon island* OR norfolk island* OR somali* OR sri lanka* OR ceylon* OR saint kitts OR st kitts OR kittian* OR nevisian* OR saint lucia* OR st lucia* OR saint vincent* OR st vincent* OR vincentian* OR grenadine* OR sudan* OR surinam* OR syria* OR tajik* OR tadjik* OR tadzhik* OR tanzania* OR tanganyika* OR thai* OR timor leste* OR east timor* OR timorese* OR togo OR togoles* OR "togo's" OR tonga* OR trinidad* OR tobago* OR tunisia* OR turkiy* OR turkey* OR turk OR turks OR turkish OR turkmen* OR uganda* OR ukrain* OR uruguay* OR uzbek* OR vanuatu* OR new hebrides* OR venezuela* OR vietnam* OR viet nam* OR yemen* OR yugoslav* OR zambia* OR zimbabwe* OR rhodesia* OR arab* countr* OR middle east* OR global south OR sahara* OR subsahara* OR magreb* OR maghrib* OR west indies* OR caribbean* OR central america* OR latin america* OR south america* OR asia central OR central asia* OR asia northern OR north asia* OR northern asia* OR asia southeastern OR southeastern asia* OR south eastern asia* OR southeast asia* OR south east asia* OR asia western OR west asia* OR western asia* OR europe eastern OR east europe* OR eastern europe* OR developing countr* OR developing nation* OR developing population* OR developing world OR less developed countr* OR less developed nation* OR less developed world OR lesser developed countr* OR lesser developed nation* OR lesser developed world OR under developed countr* OR under developed nation* OR under developed world OR underdeveloped countr* OR underdeveloped nation* OR underdeveloped world OR middle income countr* OR middle income nation* OR middle income population* OR low income countr* OR low income nation* OR low income population* OR lower income countr* OR lower income nation* OR lower income population* OR underserved countr* OR underserved nation* OR underserved population* OR under served population* OR under served nation* OR under served population* OR deprived countr* OR deprived population* OR high burden countr* OR high burden nation* OR countdown countr* OR countdown nation* OR poor countr* OR poor nation* OR poor population* OR poor world OR poorer countr* OR poorer nation* OR poorer population* OR poorer world OR developing econom* OR less developed econom* OR underdeveloped econom* OR under developed econom* OR middle income econom* OR low income econom* OR lower income econom* OR low gdp OR low gnp OR low gross domestic OR low gross national OR lower gdp OR lower gnp OR lower gross domestic OR lower gross national OR lmic OR lmics OR third world OR lami countr* OR transitional countr* OR emerging econom* OR emerging nation*)
